# Effectiveness of synthetic vs. autologous ligaments in anterior cruciate ligament reconstruction with remnant preservation: a retrospective cohort study

**DOI:** 10.3389/fspor.2026.1765712

**Published:** 2026-03-20

**Authors:** Xing Liu, Fengping Weng, Aihua Liu, Shudong Huang, Pengxin Zhang, Ming Tang, Chenghao Xiang

**Affiliations:** 1 Department of Joint Surgery Diagnosis and Treatment Center, Enshi Tujia and Miao Autonomous Prefecture Central Hospital, Enshi City, Hubei, China; 2 Health Science Center, Hubei Minzu University, Enshi City, Hubei, China; 3 Department of Sports Medicine, Wuhan Fourth Hospital, Wuhan City, China

**Keywords:** anterior cruciate ligament reconstruction, autograft, comparative effectiveness, hamstring tendon, LARS, remnant preservation, synthetic ligament

## Abstract

**Objective:**

This study aimed to evaluate the comparative efficacy of the Ligament Advanced Reinforcement System (LARS) artificial ligament vs. autologous hamstring tendons in patients undergoing anterior cruciate ligament (ACL) reconstruction with remnant preservation, with particular focus on proprioceptive recovery, joint stability, functional outcomes, return-to-sport timelines, and complication profiles during early to mid-term follow-up.

**Methods:**

We conducted a retrospective cohort analysis of 162 consecutive patients who underwent arthroscopic ACL reconstruction with remnant preservation between January 2020 and June 2023. Participants were stratified into two treatment groups: the LARS cohort (*n* = 70) and the autograft cohort (*n* = 92). Baseline demographic and clinical characteristics demonstrated comparability between groups. Standardized assessments were performed at 3, 6, 12, and 24 months postoperatively, including evaluation of proprioceptive threshold (TDPM), anterior knee stability (Ligs Digital Ligament Assessment Device), functional capacity (Lysholm score, IKDC subjective score, Tegner activity scale), return-to-sport progression, and complication incidence.

**Results:**

The autograft group exhibited significantly superior proprioceptive recovery, as evidenced by lower TDPM values at both 6 and 12-month intervals (*P* < 0.05). Enhanced anterior stability was observed in the LARS cohort at the 3-month assessment (*P* < 0.001), though this advantage diminished by 12 months. Conversely, the autograft group demonstrated significantly better patient-reported outcomes on both Lysholm and IKDC scales at the 12-month follow-up (*P* < 0.05). While the LARS group achieved higher return-to-sport rates at 12 months, the autograft group showed superior long-term sports participation at 24 months. The autograft cohort experienced 4 cases of Cyclops lesions, whereas no such complications were documented in the LARS group.

**Conclusion:**

Our findings suggest distinct temporal advantage patterns between the two graft types. The LARS artificial ligament may provide superior early stabilization and a quicker return to athletic activities in the short term, while autologous hamstring tendons appear to offer more favorable long-term functional outcomes, proprioceptive recovery, and sustained sports participation. These observations support the hypothesis that clinical decision-making should incorporate individual patient factors. Additionally, the remnant tensioning technique may offer potential benefits in reducing complications, though this requires further investigation to confirm causality.

## 1 Introduction

Anterior cruciate ligament (ACL) rupture represents a frequent orthopedic injury with an estimated incidence affecting approximately 1 in 3,000 individuals annually, substantially compromising athletic performance and quality of life (1). The evolution of surgical techniques has led to increased interest in ACL reconstruction with remnant preservation. This approach aims to conserve the native ligament's mechanoreceptors and vascular supply, potentially enhancing graft revascularization, neural regeneration, and functional recovery (2). Despite these theoretical advantages, the optimal graft selection for remnant-preserving procedures remains debated. Autologous hamstring tendons, while demonstrating favorable biocompatibility and durable outcomes, undergo a critical “ligamentization” phase lasting 6–12 months characterized by transient mechanical vulnerability. Additionally, harvest-site morbidity remains a concern, with saphenous nerve injury documented in 8%–12% of cases (3). As a synthetic alternative, the third-generation LARS (Ligament Advanced Reinforcement System) prosthesis offers immediate high tensile strength exceeding 2000N, enables early rehabilitation, eliminates donor-site complications, and potentially expedites sports resumption (4, 5).

Notwithstanding these benefits, the LARS ligament carries specific risks. Literature documents associations with inflammatory synovitis, foreign body responses, and delayed mechanical failure (6, 7). Histological assessments frequently demonstrate multinucleated giant cell infiltration and limited fibrovascular incorporation at the implantation site, potentially compromising tendon-bone integration (8). Long-term surveillance data reveal failure rates reaching 33.3% (6, 9), with emerging evidence suggesting possible correlation with accelerated osteoarthritis development (10). Whether remnant preservation techniques can modulate these risks through enhanced biological environment or tension regulation—particularly regarding Cyclops lesion formation—requires further investigation through robust clinical studies.

Current academic discourse predominantly addresses standard ACL reconstruction, with comparative analyses of graft performance in remnant-preserving settings remaining underrepresented. Significant knowledge gaps persist regarding recovery patterns, proprioceptive restoration kinetics, and Return to Sport (RTS) readiness in this specific surgical context (11, 12). We postulate that under remnant-preserving conditions, the LARS cohort may exhibit superior initial stability and accelerated RTS attributable to its inherent mechanical advantages. In contrast, autograft recipients might achieve enhanced long-term proprioception and patient-reported outcomes through superior biological integration.

This investigation employs a retrospective cohort methodology to evaluate the clinical performance of LARS artificial ligaments vs. autologous hamstring tendons in remnant-preserving ACL reconstruction. Primary analytical focus encompasses postoperative proprioception, joint stability, functional metrics (Lysholm, IKDC), complication profiles, and RTS timelines. Additionally, we assess the efficacy of the “remnant tensioning technique” as a potential preventive measure against Cyclops lesion formation, thereby contributing evidence-based insights for surgical decision-making.

## 2 Methods

### 2.1 Study design

This investigation employed a retrospective cohort design that consecutively enrolled patients undergoing arthroscopic anterior cruciate ligament reconstruction with remnant preservation at our institution between January 2020 and June 2023. We systematically stratified participants into two distinct treatment groups according to the specific surgical approach utilized: the LARS (Ligament Advanced Reinforcement System) cohort and the Autologous Hamstring Tendon cohort. In the LARS group, surgeons performed ligament reconstruction using the synthetic LARS prosthesis and achieved secure fixation at both femoral and tibial attachment sites through titanium interference screws. The autograft group received reconstruction using a quadrupled autologous graft harvested from the semitendinosus and gracilis tendons, where surgeons employed an adjustable loop button for femoral fixation and utilized a bioabsorbable interference screw for tibial fixation. The research protocol received formal approval from our institution's Ethics Review Committee. Please refer to the ethical approval form, and we obtained comprehensive written informed consent from all participants before their inclusion in the study.

### 2.2 Inclusion and exclusion criteria

Our inclusion criteria encompassed: (1) patients with radiologically and clinically confirmed unilateral complete ACL rupture; (2) cases with arthroscopic verification of preserved ligament remnant measuring ≥50% of the original ligament length; (3) individuals within age parameters restricted to 18–45 years; (4) patients with documented pre-injury activity level meeting or exceeding Tegner level 5, which indicates regular participation in high-demand sports involving jumping, cutting, and pivoting maneuvers.

Our exclusion criteria comprised: (1) patients presenting with complex multi-ligament knee injuries involving the posterior cruciate ligament, medial collateral ligament, or other stabilizing structures; (2) cases with arthroscopically identified femoral or tibial chondral lesions classified as Outerbridge grade III or higher; (3) individuals demonstrating significant lower extremity malalignment manifesting as varus or valgus deformity exceeding 5 degrees; (4) patients exhibiting concurrent meniscal pathology graded as III or chondral damage classified as grade II-IV.

### 2.3 Surgical technique

All surgical interventions were performed by a consistent team of experienced orthopedic surgeons who utilized a standardized arthroscopic approach through anteromedial portals. The technical protocol incorporated three fundamental components that surgeons implemented sequentially:

First, the remnant preservation protocol involved meticulous arthroscopic debridement of tibial and femoral ACL remnants that surgeons performed with particular attention to preserving the integrity of the synovial sheath envelope. Better stabilization and coverage over the graft can be achieved using absorbable sutures or anchor fixation techniques, thereby maintaining vascular supply and neural innervation to the residual tissue.

Second, anatomic tunnel positioning required precise bone tunnel placement that surgeons considered critical to surgical success. The surgical team strategically positioned the tibial tunnel at the anatomic center of the posterior fiber bundle within the preserved remnant, while they located the femoral tunnel at the isometric point within the native femoral footprint utilizing an over-the-top positioning technique. Surgeons conducted continuous dynamic arthroscopic assessment throughout the procedure to ensure absence of graft impingement during the complete range of knee motion, including extension, flexion, and rotational movements.

**Figure 1:**
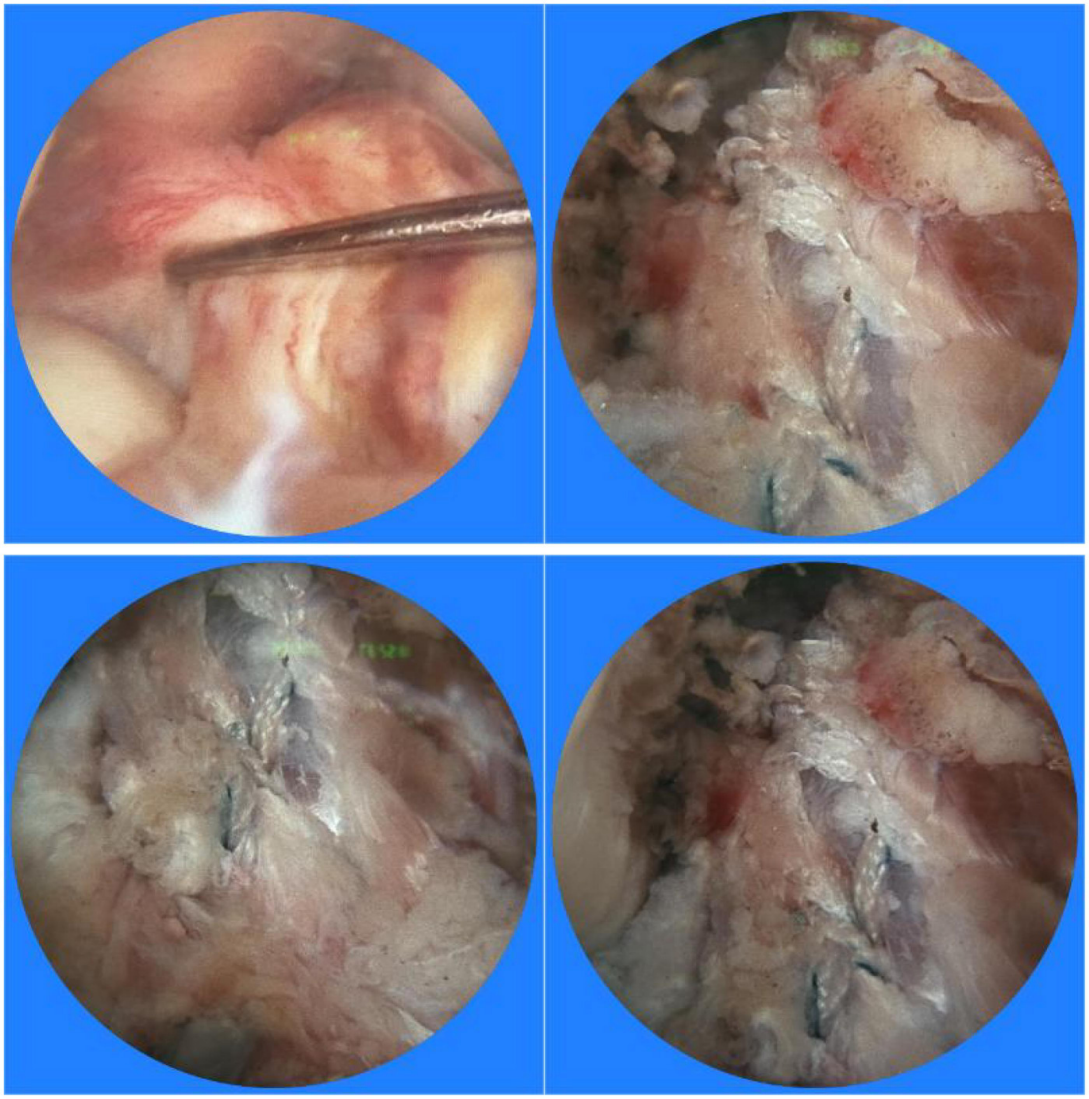
Intraoperative arthroscopic images of the autologous ligament group.

**Figure 2:**
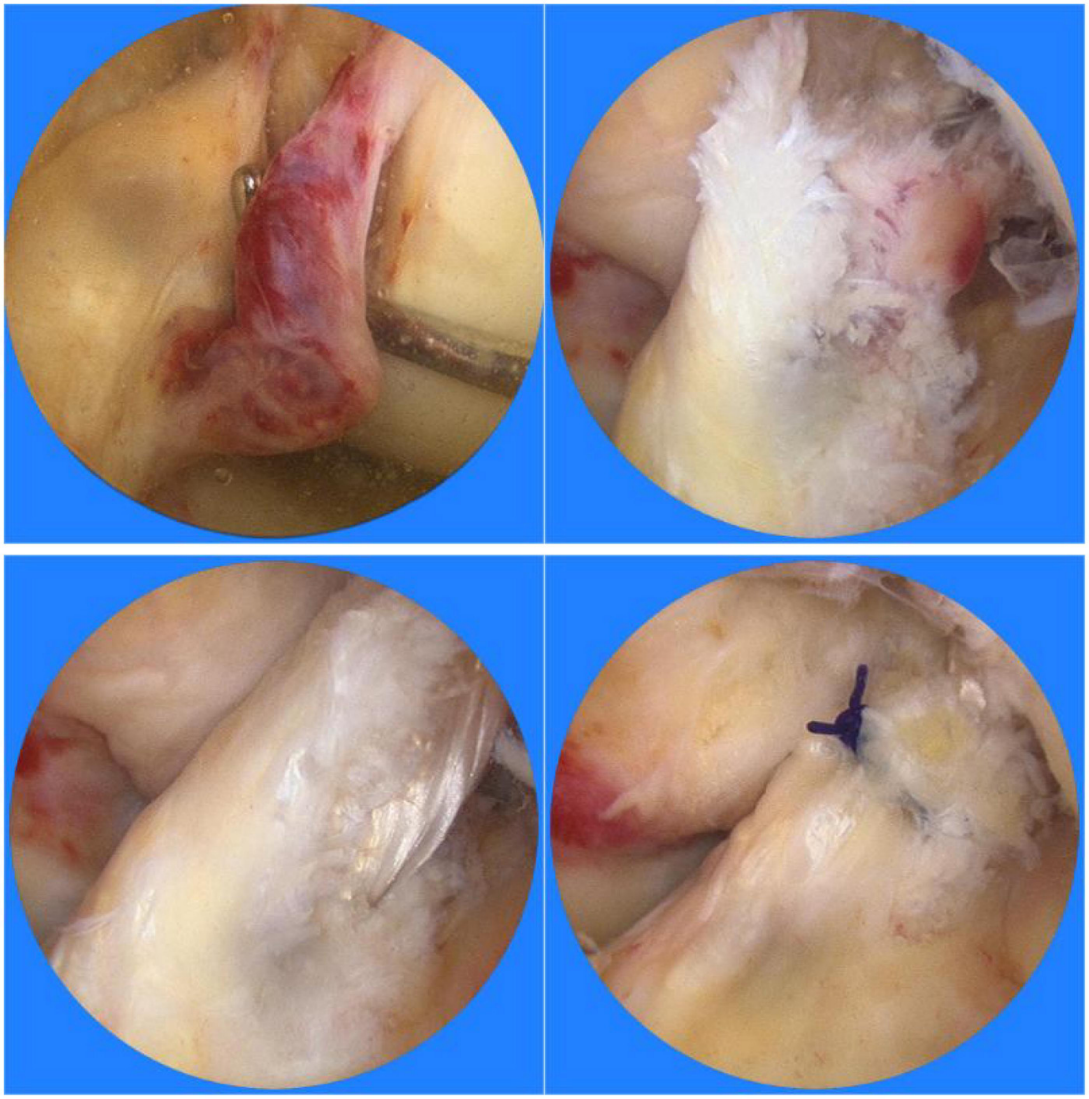
Intraoperative arthroscopic images of the artificial ligament group.

Third, the remnant tensioning maneuver was performed following graft passage but preceding final fixation, during which surgeons carefully tensioned the preserved ligament remnant using a specialized arthroscopic probe to achieve optimal apposition against the graft surface without excessive compression. This technique aimed to promote biological integration while minimizing the risk of cyclops lesion formation. The intraoperative images of the two groups are presented in Figures 1, 2, respectively.

### 2.4 Rehabilitation protocol

We implemented postoperative rehabilitation programs that were specifically tailored according to graft type characteristics, with standardized protocols strictly enforced by a dedicated team of physical therapists to minimize variability:

For the LARS cohort, surgeons and physical therapists capitalized on the inherent immediate structural strength of the synthetic ligament by implementing an accelerated rehabilitation pathway. This protocol initiated immediate protected weight-bearing with brace immobilization, progressed to full weight-bearing ambulation within a two-week timeframe, and introduced controlled non-contact athletic activities at the three-month postoperative interval.

For the autograft cohort, the medical team implemented a more conservative approach to protect the biological incorporation process by maintaining partial weight-bearing status for the initial six-week period. The rehabilitation team gradually introduced running activities at six months postoperatively and permitted full-contact sports participation only after comprehensive medical evaluation at the nine-month milestone.

All patients were required to attend weekly rehabilitation follow-up visits to ensure compliance, and compliance rates were recorded as a covariate in the statistical analysis.

### 2.5 Outcome assessment parameters

Our research team conducted comprehensive patient evaluations at three key timepoints: preoperatively, six months postoperatively, and twelve months postoperatively. We established primary outcome measures that focused on proprioceptive recovery and anterior knee stability characteristics, while secondary endpoints included functional capacity assessments, return-to-sport timelines, and complication profiles.

1. Proprioceptive Assessment: Knee joint kinesthesia was evaluated using the Threshold to Detection of Passive Movement (TDPM) protocol (11). To eliminate extraneous sensory cues, participants wore sound-attenuating earplugs, visual occlusion goggles, and knee-high stockings. Passive knee extension was administered via an isokinetic dynamometer at an angular velocity of 1°/s, starting from initial flexion angles of 15°, 30°, and 45°. Participants were instructed to signal immediately upon detecting joint motion, at which point the device was halted. The angular disparity between the starting and stopping positions was recorded. Three consecutive trials were performed at each angle, with the mean value used for subsequent analysis. It should be noted that TDPM has inherent limitations: it relies on subjective patient reporting, measures only slow passive motion detection, and may not fully reflect dynamic proprioceptive function required for daily activities or sports.
2. Anterior Knee Stability Evaluation: Side-to-side differences in anterior tibial translation (ATT) were quantified using the Ligs Digital Ligament Assessment Device under a 135N applied force at 30° and 90° of knee flexion. For measurements at 30° flexion, the tested knee was positioned on the device's specialized supports with maintained slight external rotation (15°) to ensure muscular relaxation. Patellar stabilization was achieved using two positioning pads over the patella and tibial tubercle to maintain proper trochlear engagement. The instrument was calibrated to zero following each force release, with accuracy verified within ±0.5 mm. After calibration, triplicate measurements were obtained and averaged. The identical protocol was repeated at 90° knee flexion to assess posterior tibial translation.
3. Functional Outcome Measures: Three validated instruments were employed: the Lysholm Knee Score, the International Knee Documentation Committee (IKDC) Subjective Knee Form, and the Tegner Activity Scale (13). The Lysholm score encompasses eight domains: pain, instability, locking, swelling, limping, stair climbing, squatting, and use of supportive devices, with total scores ranging from 0 to 100 (higher scores indicating better function). The IKDC evaluation utilizes a structured questionnaire assessing pain intensity, swelling characteristics, range-of-motion limitations, activities of daily living, sports participation, and quality of life impact. The Tegner scale employs a 10-level grading system, ranging from 0 (complete sedentary status due to knee problems) to 10 (participation in elite competitive sports), providing a continuum across recreational, occupational, and athletic activities. For all instruments, elevated scores correspond to superior knee-related function.
4. Structural Integrity Assessment: Cyclops lesions were identified through magnetic resonance imaging at the 12-month postoperative interval. Characteristic radiographic features included an oval-shaped mass anterior to the tibial ACL insertion, demonstrating low signal intensity on T1-weighted sequences and intermediate or heterogeneous signal on T2-weighted/PD-weighted images (reflecting its fibrovascular composition distinct from hypointense scar tissue). Associated graft deformation due to mass effect was documented when present.
5. RTS Metrics: Return-to-Sport was defined and assessed in accordance with the International Olympic Committee (IOC) consensus statement on RTS after ACL reconstruction (14), incorporating both subjective and objective functional criteria. Objective readiness-to-sport testing included strength symmetry indices, hop tests, and neuromuscular control assessments to comprehensively evaluate the patients' physical readiness for sports participation.
  5.1. Functional Criteria for RTS Clearance: Patients were deemed eligible for RTS only if they met all of the following: ① Restoration of full knee range of motion (0° extension to ≥120° flexion); ② Lysholm score ≥85 points; ③ IKDC subjective score ≥80 points; ④ Tegner activity scale score ≥ pre-injury level minus 1; ⑤ No side-to-side difference in anterior tibial translation (ATT) > 2 mm (assessed by Ligs Digital Ligament Assessment Device); ⑥ Strength symmetry indices of the knee flexors and extensors ≥90% compared to the contralateral limb, measured by isokinetic dynamometer at 60°/s and 180°/s angular velocities; ⑦ Passing dynamic stability and neuromuscular control tests (single-leg hop test, triple hop test, crossover hop test, and single-leg balance test) with symmetry ≥90% compared to the contralateral limb, where the single-leg balance test required maintaining static balance for ≥30 s with eyes closed.
  5.2. RTS Status Classification: ① Low-intensity contact sports (e.g., jogging, swimming, cycling) were documented at 12 months; ② Pre-injury level sports (including high-demand activities involving jumping, cutting, and pivoting) were confirmed at 24 months.
  5.3. Assessment Timing: Formal RTS evaluations were conducted at 12 and 24 months postoperatively by independent physical therapists strictly blinded to the graft type (LARS vs. autograft) and patient grouping information to eliminate assessor bias. RTS status was verified through a combination of standardized structured interviews, complete medical records review, and objective functional performance testing (strength symmetry tests, hop tests, neuromuscular control assessments). Additionally, we collected and adjusted for potential confounding variables affecting RTS outcomes, including patient compliance with rehabilitation protocols, pre-injury sports level, and postoperative complication occurrence, using multivariate logistic regression analysis to minimize selection bias and confounding effects in intergroup comparisons.

### 2.6 Statistical analytical methods

We performed data analysis utilizing the R statistical computing environment and expressed continuous variables as mean values accompanied by standard deviation measurements. Our analytical approach employed independent samples t-test methodology for intergroup comparisons, while longitudinal assessments utilized repeated-measures analysis of variance (ANOVA). We presented categorical data as frequency distributions with corresponding percentages and analyzed them using chi-square or Fisher's exact tests as statistically appropriate. For temporal analysis of return-to-sport outcomes, we conducted evaluations through Kaplan–Meier survival curve methodology. The distribution patterns of complication types were visualized using a radar chart, which graphically displays the incidence rates of different complications (including Cyclops lesion, graft rupture, and intra-articular infection) for each group simultaneously. We established a predetermined threshold of *p* < 0.05 (two-tailed) to determine statistical significance for all analytical comparisons.

## 3 Results

### 3.1 Baseline characteristics

A total of 162 eligible patients were included in this study, with 70 patients assigned to the LARS group and 92 patients to the Autograft group. Comparative analysis of baseline demographic and clinical characteristics—including age, sex, body mass index (BMI), time from injury to surgery, operative side, meniscal injury, and chondral damage—revealed no statistically significant differences between the two groups (all *P* > 0.05), confirming their comparability. Detailed baseline data are summarized in Table 1.

**Table 1:** Baseline characteristics.

Characteristic	LARS Group (*n* = 70)	Autograft Group (*n* = 92)	Statistical Value	*P*-value
Demographic Data				
Age (years)	29.5 ± 5.2	30.1 ± 4.8	t = 0.658	0.512
Gender (Male/Female)	38/22	35/25	*χ*^2^ = 0.321	0.571
BMI (kg/m^2^)	23.8 ± 2.5	24.1 ± 2.3	t = 0.712	0.478
Time-related Factors				
Injury-to-surgery time (weeks)	15.36 ± 5.11	16.93 ± 4.73	t = 1.234	0.219
Surgical Details				
Operative side (Left/Right)	28/42	33/59	*χ*^2^ = 0.456	0.499
Associated Injuries				
Meniscal injury (Yes/No)	42/28	56/36	*χ*^2^ = 0.087	0.768
Chondral damage (Yes/No)	28/42	26/66	*χ*^2^ = 2.891	0.089
Preoperative Functional Scores				
Lysholm score	48.3 ± 8.7	49.1 ± 9.2	t = 0.567	0.572
IKDC subjective score	45.6 ± 7.9	46.2 ± 8.3	t = 0.432	0.666
Tegner activity level	2.3 ± 0.8	2.4 ± 0.7	t = 0.789	0.431
Stability Assessment				
Preoperative ATT at 30° (mm)	5.26 ± 1.74	5.19 ± 1.67	t = 0.276	0.783
Preoperative ATT at 90° (mm)	4.43 ± 1.82	4.55 ± 1.74	t = −0.436	0.663

### 3.2 Proprioception outcomes

**Figure 3:**
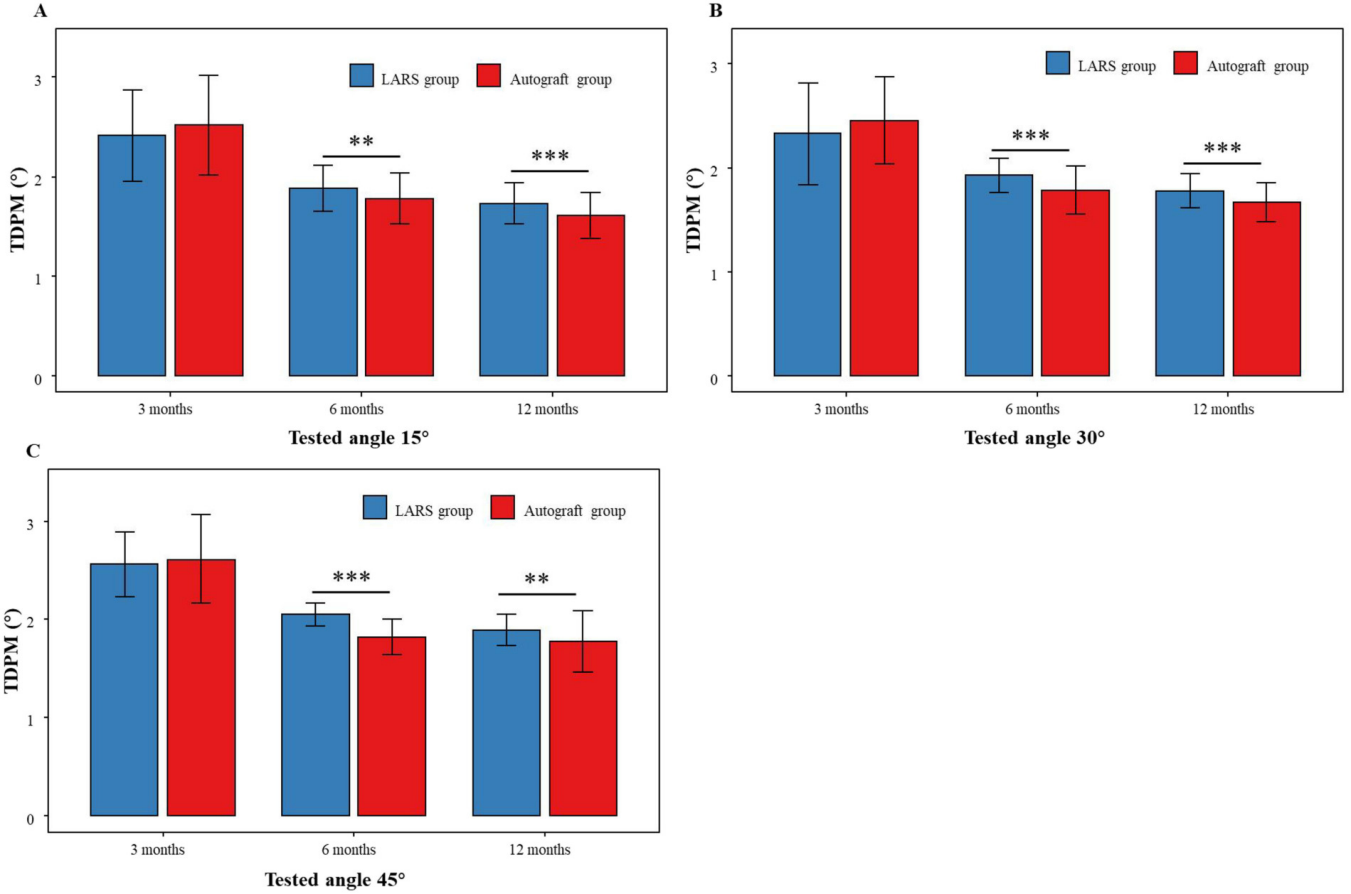
TDPM values at different knee angles and follow-up time points. **(A-C)** Grouped bar charts comparing TDPM values for LARS group and Autograft group at 3, 6, and 12 months at tested angles 15°, 30°, and 45°, respectively. **(A)** 3 months, **(B)** 6 months, **(C)** 12 months. Significant differences are marked with asterisks, indicating lower TDPM values in the Autograft group. Error bars represent standard deviation.

At the 3-month follow-up, no significant differences in the TDPM were observed between the two groups at 15°, 30°, or 45° of knee flexion. However, at the 6-month and 12-month assessments, significant reductions in TDPM values were noted in both groups at all three angles. Moreover, the Autograft group exhibited significantly lower TDPM values than the LARS group at these later time points, suggesting better detection of slow passive knee motion (a specific aspect of proprioception) in the Autograft group. The TDPM values across different knee angles and follow-up intervals are illustrated in Figure 3.

### 3.3 Anterior knee stability

At 3 months postoperatively, the side-to-side difference in ATT, as measured by the Ligs Digital Ligament Assessment Device, was significantly smaller in the LARS group than in the Autograft group at both 30° and 90° of knee flexion, demonstrating better early stability in the LARS group. However, at the 24-month follow-up, the ATT values were comparable between the two groups, with no statistically significant differences observed. The detailed results of anterior knee stability measurements are presented in Table 2.

**Table 2:** Anterior knee stability (ATT, mm).

Time	Tested angle	Group	n	ATT	t	*P*-value
Pre-operation	30°	LARS Group	70	5.26 ± 1.74	0.276	0.783
		Autograft Group	92	5.19 ± 1.67		
	90°	LARS Group	70	4.43 ± 1.82	−0.436	0.663
		Autograft Group	92	4.55 ± 1.74		
3 months	30°	LARS Group	70	2.56 ± 0.35	−5.435	<0.001
		Autograft Group	92	2.91 ± 0.47		
	90°	LARS Group	70	2.13 ± 0.43	−3.724	<0.001
		Autograft Group	92	2.37 ± 0.39		
12 months	30°	LARS Group	70	1.83 ± 0.41	0.553	0.580
		Autograft Group	92	1.79 ± 0.53		
	90°	LARS Group	70	1.25 ± 0.29	1.283	0.201
		Autograft Group	92	1.19 ± 0.32		

### 3.4 Knee function scores

**Figure 4:**
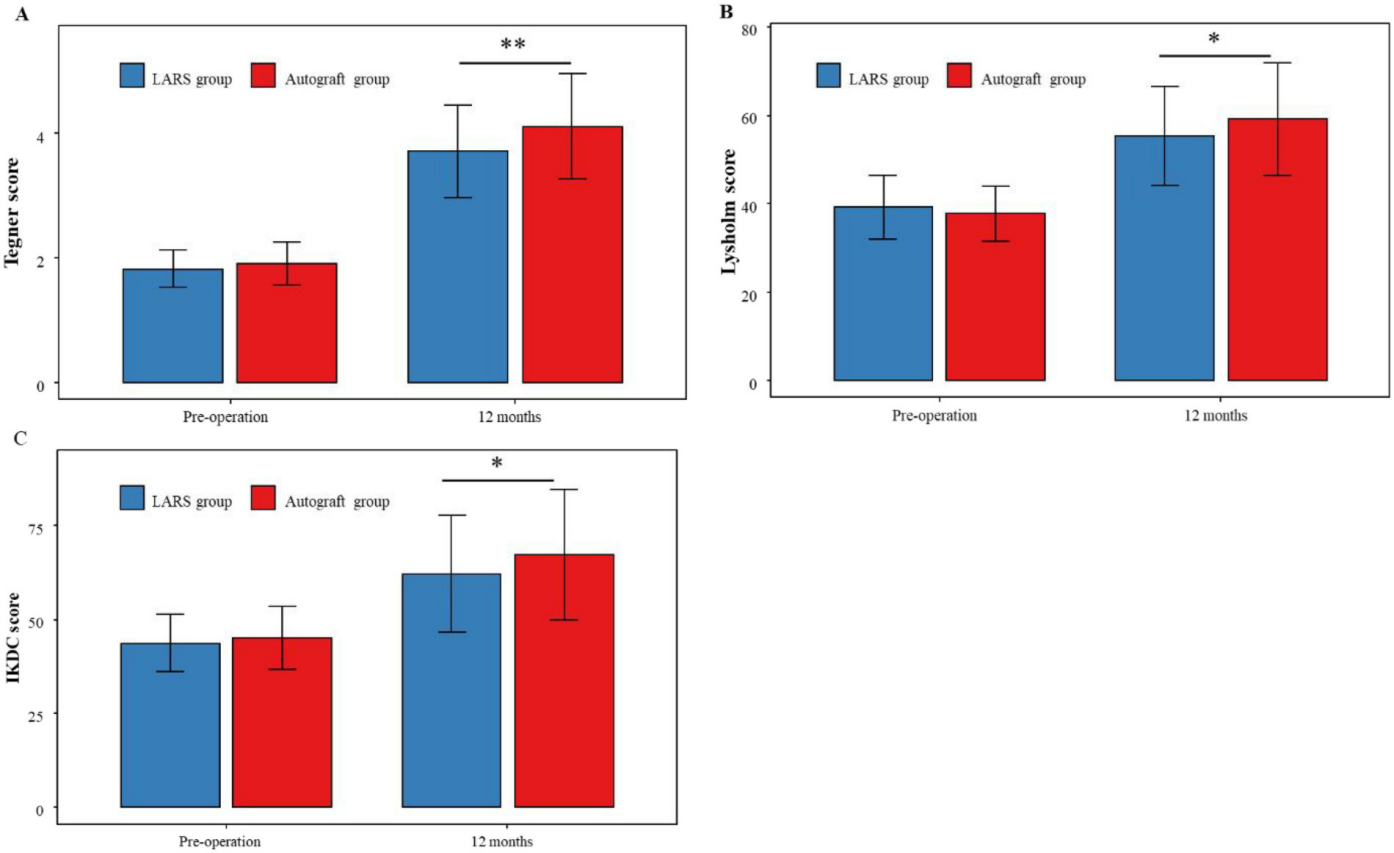
Knee functional outcomes. **(A-C)** Bar charts comparing pre-operative and 12-month postoperative scores between LARS and Autograft groups. **(A)** Tegner scores, **(B)** Lysholm scores, **(C)** IKDC scores. All scores increase at 12 months for both groups, with statistical significance indicated by asterisks. Error bars represent standard deviation.

Preoperatively, no significant differences were detected between the two groups in the Tegner activity level, Lysholm knee score, or IKDC subjective score (all *P* > 0.05). At the 12-month postoperative assessment, all three functional scores showed significant improvement from preoperative baselines in both groups (*P* < 0.05). Notably, the Autograft group achieved significantly higher scores than the LARS group on both the Lysholm and IKDC measures (all *P* < 0.05). The functional outcomes are graphically displayed in Figure 4.

### 3.5 RTS analysis

**Figure 5:**
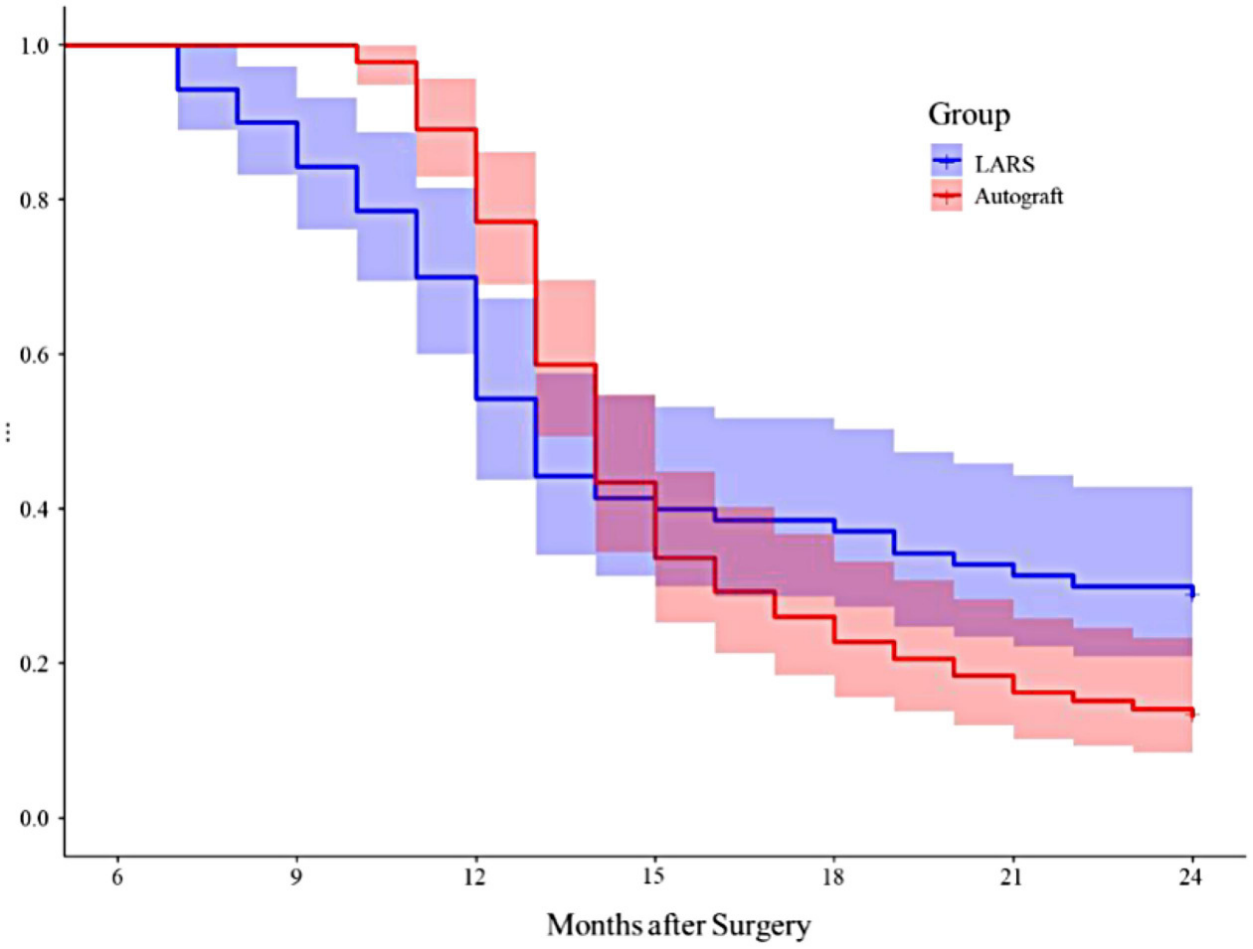
Kaplan–Meier survival curve for return to sport (RTS).

At 12 months postoperatively, a significantly higher proportion of patients in the LARS group (45.71%) had returned to low-intensity contact sports compared to the Autograft group (22.83%) (*P* < 0.001). However, with prolonged follow-up, this trend reversed by 24 months, where the percentage of patients returning to pre-injury sports levels was significantly higher in the Autograft group (86.96%) than in the LARS group (71.43%) (*P* < 0.05). The RTS analysis results are summarized in Table 3. Figure 5 presents the Kaplan–Meier survival analysis illustrating the RTS trajectories between the two groups over the 24-month follow-up period. The curves demonstrate significantly earlier RTS in the LARS group during the first postoperative year (log-rank *P* = 0.002), while the autograft group shows superior cumulative RTS rates by the end of the second year (log-rank *P* = 0.014). The divergence in survival curves after the 12-month mark reflects the temporal pattern of graft-specific recovery profiles.

**Table 3:** Return to sport (RTS) analysis.

Group	*N* (total patients)	12 months – No. (%) returned to sport	24 months – No. (%) returned to sport
LARS Group	70	32 (45.71)	50 (71.43)
Autograft Group	92	21 (22.83)	80 (86.96)
χ²		9.461	6.047
*P*		0.002	0.014

### 3.6 Complication rates

**Figure 6:**
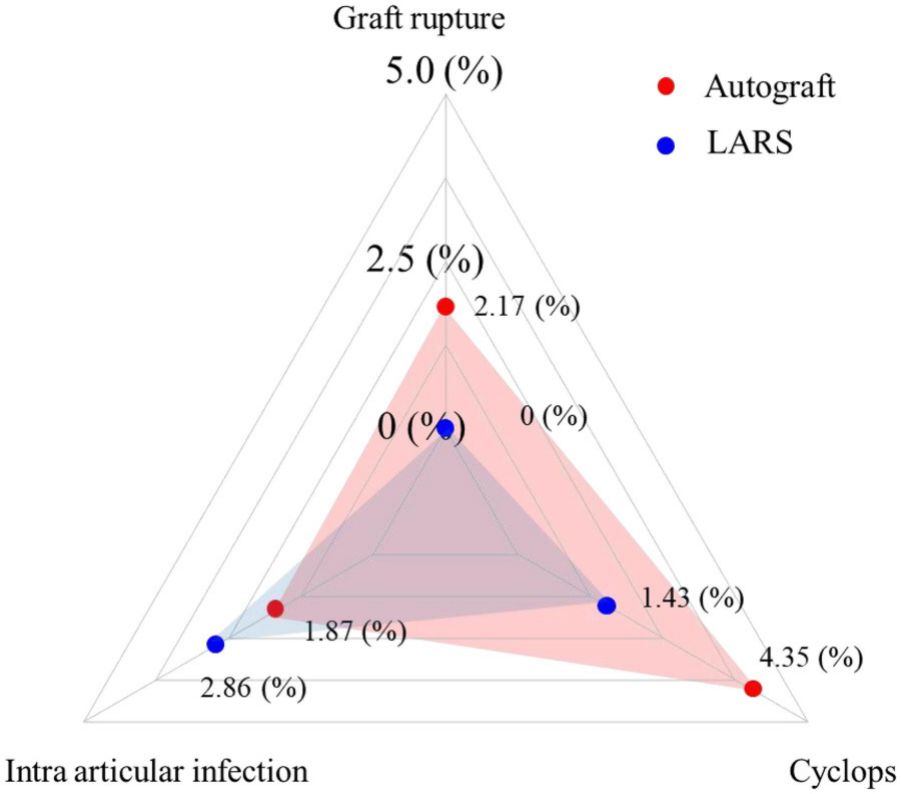
Radar chart of complication rates.

In the Autograft group, four patients (4.3%) were diagnosed with Cyclops lesions on postoperative MRI, two cases experienced graft rupture, and one patient developed an intra-articular infection. In the LARS group, one Cyclops lesion was identified, and two cases of intra-articular infection were recorded. A radar chart illustrating the distribution of complication types and frequencies is presented in Figure 6.

## 4 Discussion

This retrospective cohort study provides a comprehensive comparison of early to mid-term outcomes between LARS artificial ligament and autologous hamstring tendon in ACL remnant-preserving reconstruction. Our results demonstrate distinct clinical trajectories between the two graft types, revealing a complex interplay between biological integration capacity and mechanical properties within the context of advanced surgical preservation techniques. The differential outcomes observed across multiple assessment domains—including knee stability, functional recovery, return-to-sport timelines, and complication profiles—underscore the importance of individualized graft selection strategies based on specific patient characteristics and expectations.

However, the superior patient-reported outcomes (Lysholm and IKDC scores) achieved by the autograft group at 12 months postoperatively reaffirm the critical importance of biological integration for long-term subjective function (3), suggesting a temporal trade-off between early mechanical advantages and long-term biological incorporation.

The differential return-to-sport patterns observed provide clinically valuable insights for rehabilitation planning and patient counseling. It should be noted that the accelerated rehabilitation protocol for the LARS group may represent a potential confounding factor contributing to the higher early RTS rate at 12 months, as earlier weight-bearing and activity initiation could independently enhance functional recovery in the short term. The significantly higher RTS rate in the LARS group at 12 months (45.71% vs. 22.83%) demonstrates the combined benefit of bypassing the prolonged ligamentization process and the tailored rehabilitation strategy (5, 14), particularly beneficial for athletes with time-sensitive competitive schedules or professional commitments. However, the reversal of this trend at 24 months, with the autograft group achieving superior final RTS rates (86.96% vs. 71.43%), indicates that biological grafts ultimately provide more sustainable solutions for high-demand athletic activities independent of rehabilitation differences (11, 15). We adjusted for rehabilitation compliance in the statistical analysis, and the observed long-term RTS disparity persisted, suggesting that the intrinsic biological integration advantage of autografts is the dominant factor in sustained sports participation. This temporal pattern underscores the need for a patient-specific approach to graft selection based on individual recovery timelines, athletic goals, and long-term expectations rather than seeking universal superiority of one graft type.

Based on these findings, we propose a nuanced decision-making framework that integrates multiple patient-specific factors. For competitive athletes requiring rapid return to play, patients with limited rehabilitation tolerance, or those with specific temporal constraints, the LARS artificial ligament may offer potential advantages in early functional recovery—though patients should be counseled on potential long-term risks (16). Conversely, we hypothesize that autologous hamstring tendons may represent a more biologically compatible option for younger patients, those with open physes, revision scenarios, or individuals prioritizing long-term joint health, potentially due to a lower risk of foreign body reaction (8, 17). These proposed preferences require individualization and further validation in targeted patient cohorts. The decision-making process should also incorporate surgical factors such as remnant tissue quality and autograft availability, situations where the mechanical advantages of LARS become particularly relevant.

The complication profiles of the two graft types exhibited distinct characteristics that should be interpreted in full alignment with the study results. Specifically, the autograft group presented with a higher incidence of Cyclops lesions (4 cases, 4.3%), along with 2 cases of graft rupture and 1 case of intra-articular infection; in contrast, the LARS group had a lower Cyclops lesion rate (1 case, 1.4%) but a relatively higher intra-articular infection rate (2 cases, 2.9%).

Notably, the clinical relevance of intra-articular infections deserves explicit emphasis. All 3 infected cases (1 in autograft group, 2 in LARS group) were managed with arthroscopic debridement combined with targeted antibiotic therapy for 6–8 weeks. Among them, the 2 infected patients in the LARS group experienced a 4–6 week delay in return to low-intensity sports, though no long-term sequelae such as joint stiffness or graft failure were observed during the 24-month follow-up. Potential risk factors for infection in the LARS group may include the foreign body nature of the synthetic graft, which could induce mild local inflammatory responses that increase susceptibility to bacterial colonization. Meanwhile, the graft rupture cases in the autograft group were associated with premature return to high-intensity sports before completion of the ligamentization phase, highlighting the importance of strict adherence to rehabilitation protocols for biological graft recipients.

The tensioned remnant technique may contribute to reducing the incidence of Cyclops lesions and synovitis in both groups, but it cannot eliminate the device-specific risks of synthetic grafts or the biological vulnerabilities of autologous grafts during the ligamentization period. The technique's ability to create optimal tissue apposition without excessive tension likely contributes to enhanced biological integration while preventing complications associated with redundant or improperly tensioned remnant tissue. Future investigations should explore the combination of this technique with biological augmentation strategies, such as platelet-rich plasma applications or stem cell technologies, to further optimize outcomes and potentially accelerate the integration process for both graft types.

Several methodological considerations warrant careful acknowledgment when interpreting our results. The 24-month follow-up period, while providing valuable intermediate-term data, is a major limitation of the present study. It cannot address long-term concerns regarding synthetic graft durability, particularly beyond the 10-year horizon, including late-stage mechanical failure, progressive joint degeneration, and chronic complications (e.g., delayed synovitis, osteoarthritis progression) that may manifest decades after implantation. The retrospective design, despite rigorous statistical adjustment for known confounders and baseline matching, carries inherent risk of selection bias and unmeasured confounding. Additionally, our study did not investigate the potential synergistic effects of biological enhancement technologies—such as stem cell coatings or growth factor treatments—which represent an important direction for future research aimed at optimizing graft integration and performance. While our sample size provided adequate statistical power for primary outcome assessments, larger multicenter prospective studies with extended follow-up periods (≥10 years) are urgently needed to establish definitive clinical guidelines and evaluate rare but clinically significant long-term complications of LARS artificial ligaments, thereby confirming the long-term durability of these implants. Additionally, our study did not investigate the potential synergistic effects of biological enhancement technologies—such as stem cell coatings or growth factor treatments—which represent an important direction for future research aimed at optimizing graft integration and performance. While our sample size provided adequate statistical power for primary outcome assessments, larger multicenter prospective studies with extended follow-up periods (≥10 years) are urgently needed to establish definitive clinical guidelines and evaluate rare but clinically significant long-term complications of LARS artificial ligaments, thereby confirming the long-term durability of these implants.

Notably, the minimal clinically important difference (MCID) for TDPM in ACL reconstruction patients has been reported to be 1.2°–1.5° at 6–12 months postoperatively (18). In our study, the mean TDPM difference between the autograft and LARS groups was 1.3° at 6 months and 1.6° at 12 months, indicating that the observed between-group disparity exceeds the MCID and thus possesses clinical relevance. However, the correlation between TDPM improvement and actual functional performance in daily activities or sports remains to be verified by further studies.

Internal Bracing—a technique that preserves the entire tibial remnant and augments graft stability—provides valuable insights into the independent effect of remnant preservation on proprioception. Existing studies on this technique have shown inconsistent proprioceptive outcomes: Wen et al. (19) conducted a cohort study of 89 patients and found that while Internal Bracing achieved superior early joint stability (similar to the LARS group in our study), its proprioceptive outcomes (assessed via TDPM and joint position sense tests) at 6 and 12 months were not significantly better than those of standard remnant-preserving reconstruction. Similarly, another retrospective study (20) reported that Internal Bracing patients exhibited improved proprioception only when combined with autologous grafts, but not with synthetic grafts—consistent with our finding that biological grafts are more likely to exploit the biological potential of remnant preservation.

## 5 Conclusion

In ACL reconstruction with remnant preservation, the choice between LARS artificial ligament and autologous hamstring tendon involves balancing temporally distinct advantage profiles. The LARS graft demonstrates superior early stability and accelerated return to sport, while autologous hamstring tendons provide better long-term functional outcomes, improved detection of slow passive knee motion (as measured by TDPM), and higher ultimate return-to-sport rates. Due to TDPM's inherent limitations, this does not equate to superior overall proprioceptive function. Clinical decision-making should be individualized based on patient-specific factors including age, activity demands, temporal constraints, and biological considerations, with the remnant tensioning technique representing a promising surgical refinement for complication reduction. These findings contribute to the development of evidence-based, patient-centered approaches to graft selection in modern ACL surgery, emphasizing the importance of matching graft properties to individual patient needs and expectations.

## Data Availability

The data analyzed in this study is subject to the following licenses/restrictions: The datasets generated and analyzed during this study are not publicly available due to privacy and ethical restrictions imposed by the ethics committee and the hospital, as they contain confidential patient health information. However, they are available from the corresponding author on reasonable request for legitimate research purposes, subject to necessary approvals. Requests to access these datasets should be directed to Xiang Chenghao, 65628325@qq.com.
